# The application of tailor-made force fields and molecular dynamics for NMR crystallography: a case study of free base cocaine

**DOI:** 10.1107/S2052252517001415

**Published:** 2017-02-15

**Authors:** Xiaozhou Li, Marcus A. Neumann, Jacco van de Streek

**Affiliations:** a Department of Pharmacy, University of Copenhagen, Universitetsparken 2, Copenhagen DK-2100, Denmark; b Avant-garde Materials Simulation Deutschland GmbH, Rosa-Luxemberg-Strasse 14, Freiburg D-79100, Germany

**Keywords:** molecular dynamics, density functional theory, crystal structure prediction, NMR crystallography, cocaine free base

## Abstract

Motional averaging has been proven to be significant in predicting the chemical shifts in *ab initio* solid-state NMR calculations, and the applicability of motional averaging with molecular dynamics has been shown to depend on the accuracy of the molecular mechanical force field. The performance of a fully automatically generated tailor-made force field (TMFF) for the dynamic aspects of NMR crystallography is evaluated and compared with existing benchmarks, including static dispersion-corrected density functional theory calculations and the COMPASS force field. The crystal structure of free base cocaine is used as an example. The results reveal that, even though the TMFF outperforms the COMPASS force field for representing the energies and conformations of predicted structures, it does not give significant improvement in the accuracy of NMR calculations. Further studies should direct more attention to anisotropic chemical shifts and development of the method of solid-state NMR calculations.

## Abstract 2

The performance of a fully automatically generated tailor-made force field in the field of NMR crystallography is evaluated and compared with existing benchmarks. The advantages and limitations of NMR crystallography with motional averaging are revealed in this study.

## 1. Introduction   

*In silico* molecular modelling methods, such as electronic structure methods and classical mechanics, have played a significant role in the elucidation of the structural and dynamic properties of molecular crystals over the past few decades (Beran, 2016 ▸; Gavezzotti, 2012 ▸; Abramov, 2016 ▸). One emerging field, denoted ‘NMR crystallography’, incorporating *ab initio* calculations with solid-state nuclear magnetic resonance (SS-NMR) spectroscopy and possibly powder X-ray diffraction (PXRD), shows a remarkable capability in the understanding of molecular crystals at the molecular level, if single crystals are difficult or impossible to obtain (Harris *et al.*, 2009 ▸; Martineau *et al.*, 2014 ▸; Ashbrook & McKay, 2016 ▸). SS-NMR spectroscopy shows the robustness necessary to handle the vast majority of samples, and the resolution is extremely high so that small differences in the electronic environment of atoms can be identified (Apperley *et al.*, 2012 ▸). It can also interpret dynamic aspects such as disorder in molecular crystals (Martineau *et al.*, 2014 ▸; Apperley *et al.*, 2012 ▸). Density functional theory (DFT)-based methods are widely used in NMR crystallography. For example, dispersion-corrected DFT (DFT-D), which is a popular and practical method that provides a compromise between accuracy and speed to reproduce the packings and energies of molecular crystals (Day *et al.*, 2009 ▸; Bardwell *et al.*, 2011 ▸; Reilly *et al.*, 2016 ▸), is used extensively for geometry optimizations (Dudenko *et al.*, 2013 ▸; Li *et al.*, 2014 ▸; Hartman & Beran, 2014 ▸; Sneddon *et al.*, 2014 ▸; Widdifield *et al.*, 2016 ▸; Watts *et al.*, 2016 ▸; Gumbert *et al.*, 2016 ▸; Folliet *et al.*, 2013 ▸), and the gauge-including projector augmented wave (GIPAW) method (Pickard & Mauri, 2001 ▸) is used to calculate magnetic shieldings for periodic systems.

DFT calculations are conducted at zero kelvin, whereas SS-NMR experiments are usually performed at ambient temperature and represent an average of space and time, thus leading to an inconsistency between experimental and theoretical predictions. Therefore, methods that introduce vibrational averaging to the system of interest have been developed and applied in a series of case studies, including introducing the effects of temperature using a perturbative expansion within the harmonic approximation (Monserrat *et al.*, 2014 ▸), classical molecular dynamics (MD) with transferable force fields (De Gortari *et al.*, 2010 ▸; Li *et al.*, 2016 ▸) or force field parameters derived from *ab initio* MD simulations (Robinson & Haynes, 2010 ▸), Born–Oppenheimer approximation-based *ab initio* MD (De Gortari *et al.*, 2010 ▸; Dračínský & Bouř, 2012 ▸; Dračínský & Hodgkinson, 2013 ▸), Car–Parrinello MD (Wegner *et al.*, 2011 ▸), path integral MD (Dračínský & Hodgkinson, 2014 ▸; Dračínský *et al.*, 2016 ▸), quantum Monte Carlo (Monserrat *et al.*, 2014 ▸) and so forth. The most popular method for integrating the thermal motion in *ab initio* NMR calculations is *ab initio* MD; however, it requires intensive computational resources to carry out a simulation on a timescale of picoseconds, and the simulation box is usually restricted to a single unit cell. Traditional classical mechanical force fields are not normally transferable to a wide spectrum of systems because they may not represent the correct potentials or configurations for systems of interest (Nemkevich *et al.*, 2010 ▸; Nyman *et al.*, 2016 ▸). The ‘tailor-made force field’ (TMFF) technique is a promising candidate for overcoming such limitations that inhibit the accuracy of SS-NMR calculations: the force field parameters are fitted against DFT-D reference data, including the packings and bonded and non-bonded interactions for individual molecules (Neumann, 2008 ▸). The technique was originally developed for crystal structure prediction (CSP) in *GRACE* (Avant-garde Materials Simulation Deutschland GmbH, Freiburg, Germany) for preliminary structure generation and conformational analysis. The latest version of *GRACE* allows us to export TMFFs to third-party MD simulation packages, providing seamless integration to investigate the dynamic behaviour of molecular crystals with high-quality force fields derived from DFT-D reference data.

Herein, we present a computational study which aims to evaluate the performance of a TMFF to average thermal effects in the calculation of SS-NMR magnetic shieldings, by identifying the correct experimental crystal structure from a list of CSP-generated candidates. In previous studies, multiple candidates from a CSP study were compared with the experimental SS-NMR pattern by means of static DFT calculations (Baias, Widdifield *et al.*, 2013 ▸; Baias, Dumez *et al.*, 2013 ▸; Salager *et al.*, 2010 ▸). Case studies of individual compounds have shown that averaging over configurations from a classical MD simulation improves the accuracy of the prediction compared with static SS-NMR calculations (Li *et al.*, 2016 ▸). In this paper, we therefore combine the two approaches and run MD simulations for each of the predicted structures, requiring over 600 SS-NMR calculations. Previous investigations revealed that the usefulness of MD averaging depends on the quality of the molecular mechanical force field (Li *et al.*, 2016 ▸; Nemkevich *et al.*, 2010 ▸). The quality of the force field is addressed by using a TMFF, *i.e.* a non-transferable force field that was parameterized from scratch for the compound of interest. A similar approach which combines DFT energy minimizations and a quantum mechanically derived force field (QMDFF) has already been applied to the calculation of electronic circular dichroism (ECD) spectra for [16]-helicene, which achieves good agreement with the experimental data (Bannwarth *et al.*, 2016 ▸).

**Figure 1:**
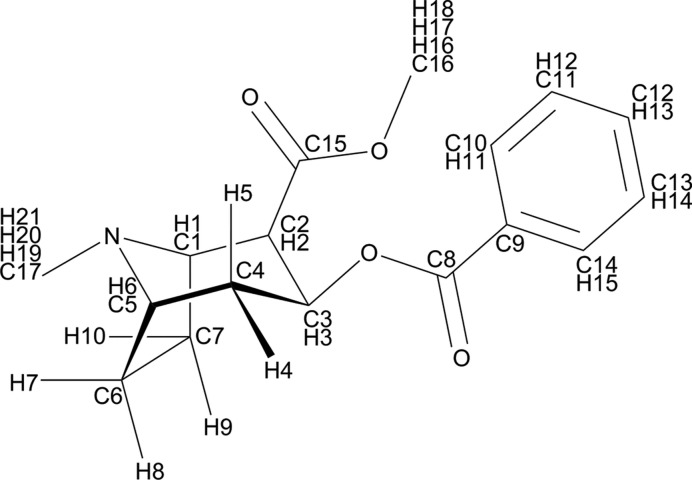
The chemical formula and atomic labels of a cocaine molecule.

The crystal structure of free base cocaine is used here as an example. Cocaine (IUPAC systematic name: methyl (1*R*,2*R*,3*S*,5*S*)-3-(benzoyloxy)-8-methyl-8-azabicyclo[3.2.1]octane-2-carboxylate; Fig. 1 ▸) is a local anaesthetic, and (−)-cocaine exists as a pure enantiomer in nature and can be extracted from coca leaves (Casale, 1987 ▸). Only one crystal structure of the free base form has been determined thus far (Hrynchuk *et al.*, 1983 ▸). Baias, Widdifield *et al.* (2013 ▸) published an NMR crystallography study for cocaine, showing that static SS-NMR calculations were able to identify the correct experimental structure when using a ^1^H spectrum, but when using a ^13^C spectrum the correct structure could not be singled out. The chemical shifts were calculated using three different approaches: static DFT-D energy minimization, motional averaging with the COMPASS force field and motional averaging with a TMFF. The deviations between the calculated and experimental isotropic chemical shifts were used to quantify the performance of these three approaches.

## 2. Methods   

### 2.1. Input   

The two-dimensional structural formula of the compound was required for the parameterization of the TMFF and the CSP. The experimental crystal structure of free base cocaine was obtained from the Cambridge Structural Database (CSD; Groom *et al.*, 2016 ▸) with reference code COCAIN10 (Hrynchuk *et al.*, 1983 ▸). The assignments of the ^1^H and ^13^C experimental chemical shifts for free base cocaine were taken from Baias, Widdifield *et al.* (2013 ▸).

### 2.2. Parameterization of the TMFF and CSP for free base cocaine   

*GRACE* (version 2.4.87) was used for the parameterization of the TMFF and CSP for free base cocaine. Both the TMFF parameterization and the CSP were fully automatic. The protocols for the TMFF parameterization and the CSP are reported in detail elsewhere (Neumann, 2008 ▸; Kendrick *et al.*, 2011 ▸, 2013 ▸). *GRACE* achieved high success rates in recent CSP blind tests (Day *et al.*, 2009 ▸; Bardwell *et al.*, 2011 ▸; Reilly *et al.*, 2016 ▸). The TMFF of free base cocaine employed for further MD simulations was parameterized in the DREIDING format (Mayo *et al.*, 1990 ▸) with van der Waals interactions described by the Lennard–Jones 9-6 (LJ 9-6) form. For the sake of completeness, we mention that *GRACE* is also able to parameterize van der Waals interactions using the Lennard–Jones 12-6 form or the exponential-6 form.

In the CSP of free base cocaine, the final lattice-energy ranking was carried out by DFT-D calculations (Kendrick *et al.*, 2013 ▸). *GRACE* employs the plane-wave DFT code *VASP* [version 5.2; University of Vienna, Austria (Kresse & Furthmüller, 1996*a* ▸,*b* ▸; Kresse & Joubert, 1999 ▸)] to carry out DFT single-point energy calculations. An in-house developed quasi-Newton algorithm was used for energy minimizations (Neumann & Perrin, 2005 ▸). The Perdew–Burke–Ernzerhof generalized gradient approximation (GGA) exchange-correlation functional (Perdew *et al.*, 1996 ▸) with the Grimme-2010 dispersion correction (Grimme *et al.*, 2010 ▸) was used for the DFT calculations, referred to as PBE-D3. A plane-wave kinetic energy cut-off of 520 eV and a Monkhorst–Pack grid (Monkhorst & Pack, 1976 ▸) with a *k*-point sampling spacing of approximately 0.07 Å^−1^ were used for the integration over the first Brillouin zone. The convergence criteria for the DFT-D lattice-energy minimizations were adopted from one of our previous studies (Neumann *et al.*, 2015 ▸).

In accordance with enantiomerically pure free base cocaine with one molecule in the asymmetric unit, the CSP was carried out using all 65 Sohncke space groups.

### 2.3. Static DFT-D energy minimizations   

The plane-wave DFT code *CASTEP* (academic version 6.1; Clark *et al.*, 2005 ▸) was used for DFT-D energy minimizations of predicted structures for static SS-NMR calculations. In this version of *CASTEP*, the Grimme-2010 (-D3) dispersion correction is not available. Thus, to keep the consistency of the protocols we used for two of our previous studies (Li *et al.*, 2014 ▸, 2016 ▸), the semi-empirical type Grimme-2006 dispersion-correction scheme (Grimme, 2006 ▸) with the PBE functional was used, referred to as PBE-D2. The protocol for the energy minimizations was described by Li *et al.* (2014 ▸) with one exception in this particular case: energy minimizations with only the hydrogen atoms allowed to move were omitted, because all the crystal structure candidates were generated from PBE-D3 calculations; the positions of the hydrogen atoms are, in most cases, more accurate than the experimental X-ray diffraction data. The convergence criteria for the energy minimizations were: change in the total energy 9.649 × 10^−4^ kJ mol^−1^, maximum force 2.895 kJ mol^−1^ Å^−1^, maximum stress tolerance 0.05 GPa, maximum atomic displacement 0.003 Å.

### 2.4. Energy minimizations with the COMPASS force field and the TMFF   

To evaluate the accuracy of the COMPASS force field and the TMFF in reproducing the energies of molecular crystals, the CSP candidates were subjected to energy minimizations with these two force fields. The corresponding lattice energies were calculated. The 26 predicted crystal structures were imported into *Materials Studio* (version 6.0; Accelrys Inc., San Diego, California, USA) and underwent energy minimizations with the COMPASS force field (Sun, 1998 ▸). The cell parameters were allowed to vary. The Ewald summation method (Ewald, 1921 ▸) was employed to consider the electrostatic and van der Waals interactions. The convergence thresholds were: 4.184 × 10^−4^ kJ mol^−1^ for the energy changes, 2.092 × 10^−2^ kJ mol^−1^ Å^−1^ for the forces, 0.005 GPa for the stresses and 5.0 × 10^−5^ Å for the atomic displacements. The energy-minimized structures with the TMFF were obtained directly from the TMFF structure generation procedure in the CSP of free base cocaine.

### 2.5. MD simulations   

The *Forcite Plus* module in *Materials Studio* was used for all the classical MD simulations with the COMPASS force field (Sun, 1998 ▸) or the TMFF for free base cocaine. Periodic boundary conditions were applied. The time step was 1 fs and the space group of all the simulation cells was *P*1 in all simulations. Similar to our previous study (Li *et al.*, 2016 ▸), a two-step approach which switched between a large and a small simulation cell was used, which allowed us to combine physically realistic large system sizes with fast SS-NMR calculation times.

Due to the computational cost of MD simulations and the subsequent SS-NMR calculations, structures with root-mean-square deviations (RMSDs) between the experimental and calculated ^13^C chemical shifts larger than three standard deviations of the mean (*i.e.* 3.1 p.p.m.) in static DFT-D calculations were not subjected to MD simulations.

#### 2.5.1. Large-cell simulations   

To use a relatively large cut-off distance for non-bonded interactions (electrostatic and van der Waals) and to lessen the self-interactions introduced by periodic boundary conditions, the CSP candidates were replicated into large supercells, each containing 576 cocaine molecules. The cut-off distance for both types of non-bonded interaction was 25 Å. Each side-to-side distance of the supercell was not less than 50 Å (twice the cut-off distance). Each supercell was first energy-minimized with the unit-cell parameters free to vary. It was then subjected to a temperature series for equilibration at 50, 150 and 300 K. The equilibration was split into three steps. First, the *NVT* ensemble and the Berendsen thermostat (Berendsen *et al.*, 1984 ▸) were used; in this step the cell parameters were fixed and the simulation time was 2.0 ps. Second, the *NPT* ensemble and the Berendsen thermostat and barostat were used for 10.0 ps. In the third step, the *NPT* ensemble was used for 10.0 ps, with the Nosé–Hoover–Langevin (NHL) thermostat (Samoletov *et al.*, 2007 ▸) to control the temperature and the Parrinello barostat (Parrinello & Rahman, 1981 ▸) to control the pressure. Finally, a 150.0 ps production run was carried out at 300 K using the NHL thermostat and the Parrinello barostat.

#### 2.5.2. Small-cell simulations   

The sizes of the large supercells are not suitable for electronic structure methods. Therefore, small-cell simulations were conducted on the basis of the corresponding large supercell simulations of the CSP candidates. The numbers of molecules in each small cell were the same as in the CSP candidate (*i.e.* a 1 × 1 × 1 unit cell), except for structure 9, the only CSP candidate that has *Z* = 1, which was replicated 2 × 2 × 2 times in order to reduce possible self-interactions. The cell parameters of the small cells were calculated by averaging the cell parameters in the 300 K production runs of the large cells. The *NVT* ensemble was used for the MD simulations of the small cells. An equilibration with the Berendsen thermostat was used for 5.0 ps, followed by a 100.0 ps production run with the NHL thermo­stat. The Ewald summation method (Ewald, 1921 ▸) was used for the description of both the electrostatic and the van der Waals interactions. To keep the numbers of molecules consistent in the SS-NMR calculations for each CSP candidate, different numbers of frames were selected from the MD trajectories for SS-NMR calculations. For *Z* = 2 and *Z* = 4 structures, 24 and 12 frames were selected from each production run of the small cell with intervals of 4.0 and 8.0 ps, respectively. For structure 9, six frames were selected with an interval of 16.0 ps. Therefore, for each predicted structure, the chemical shifts were obtained using the average over 48 calculated molecular spectra.

### 2.6. SS-NMR calculations in *CASTEP*   

Crystal structures of CSP candidates, after DFT-D energy minimizations, MD simulations with the COMPASS force field and MD simulations with the TMFF, were subjected to *ab initio* NMR calculations using the DFT-based GIPAW method (Pickard & Mauri, 2001 ▸) in *CASTEP*. Integrals taken over the first Brillouin zone were conducted on a Monkhorst–Pack grid with a *k*-point spacing not larger than 0.05 Å^−1^ and at least two *k*-points along each direction. An energy cut-off of 1200 eV was used and Vanderbilt-type ultrasoft pseudo­potentials were generated on-the-fly during the SS-NMR calculations (Yates *et al.*, 2007 ▸). The hydrogen atoms on the methyl groups undergo fast exchange at ambient temperature within the acquisition time of NMR experiments. Therefore, an averaged ^1^H chemical shift was calculated and used for the three hydrogen atoms on each methyl group for all three approaches.

The calculated isotropic magnetic shieldings were then converted to chemical shifts using the relation 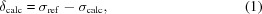
where δ_calc_ is the calculated chemical shift, σ_calc_ is the calculated isotropic shielding and σ_ref_ is the reference shielding. For each computational approach, an averaged reference shielding for all the candidates was calculated, *i.e.* the reference shieldings of the PBE-D2 energy-minimized structure, the motional averaging with the COMPASS force field and the motional averaging with the TMFF are different. Each σ_ref_ value was obtained by a linear regression between the calculated shieldings and the experimental shifts with the slope constrained to unity (Harris *et al.*, 2007 ▸).

## 3. Results and discussion   

### 3.1. The CSP   

**Figure 2:**
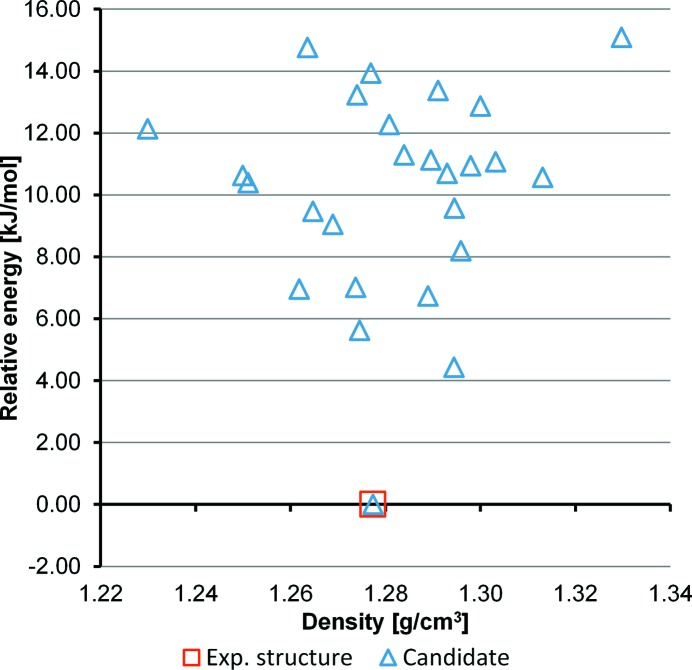
The crystal energy landscape of free base cocaine from the CSP. Each symbol represents a predicted structure. The experimental structure is indicated with a red box. The lowest lattice energy was calibrated to zero.

**Figure 3:**
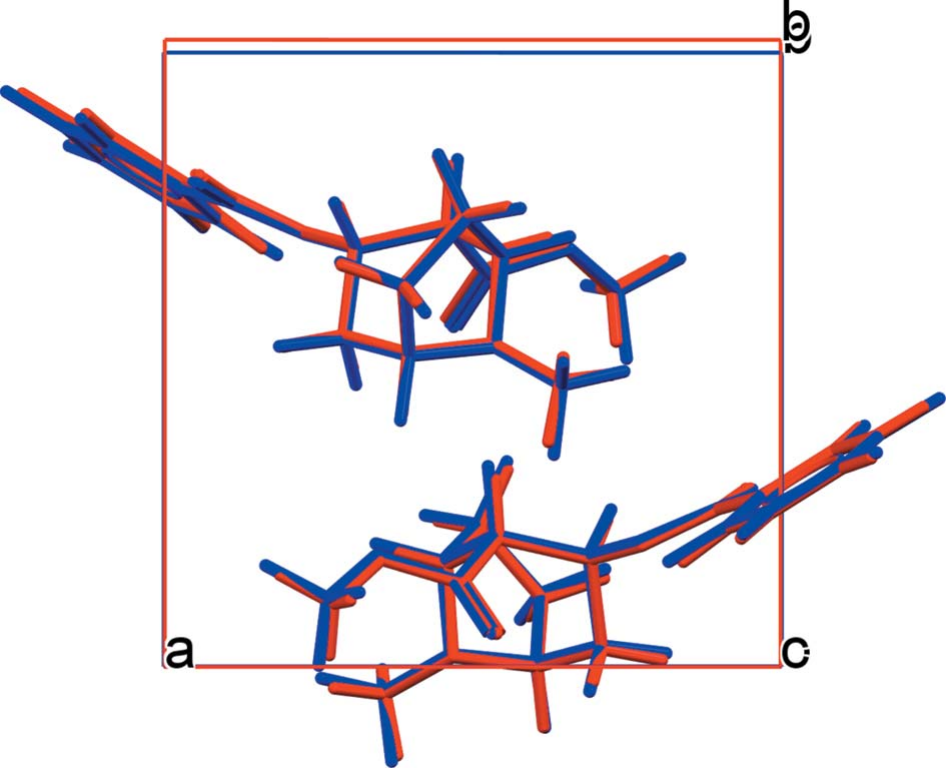
An overlay of the experimental crystal packing of free base cocaine (in red, CSD reference code COCAIN10) and structure 1 from the CSP (in blue).

Twenty-six crystal structure candidates were captured in the final list of the CSP, representing different possible polymorphs. The crystal energy landscape of CSP candidates is shown in Fig. 2 ▸, represented by plotting the calculated relative lattice energies against the densities of the candidates. The lattice energy of the lowest-energy form among the 26 candidates was calibrated to zero. The experimental form is successfully predicted with the lowest energy (rank No. 1) of all the candidates. An overlay of the experimental structure and structure 1 from the CSP is shown in Fig. 3 ▸, demonstrating the good agreement between experiment and prediction; the non-hydrogen root-mean-square Cartesian displacement (RMSCD) between the experimental structure and structure 1 is 0.0556 Å. The lattice-energy margin between structures 1 and 2 is 4.43 kJ mol^−1^, a relatively large gap indicating that additional polymorphs may be difficult to find (Habgood, 2011 ▸; Price, 2014 ▸). The vibrational contributions to the relative free energies are typically smaller than 2 kJ mol^−1^ (Nyman & Day, 2015 ▸), which, compared with the energy gap between structures 1 and 2, is a minor issue to consider. The predicted structures can be found in Table S1 in the supporting information.

Cocaine is chiral and known to have *Z*′ = 1 from the SS-NMR experiment. This reduces the range of possible packings in the CSP and thus the correct structure can be selected based solely on the crystal energy landscape; the large energy gap provides a good indication. However, the energy landscape of cocaine is rather unusual in this respect. In more complex cases all 230 space groups are used in CSPs for discovering possible polymorphs. The lattice energies of different forms are typically very similar, meaning that the energy landscapes are not informative enough to reveal the correct forms (Price, 2014 ▸). For this reason, the energy landscape of free base cocaine was not used to select the correct form from a list of candidates.

### 3.2. The energy profiles of CSP candidates: COMPASS and TMFF   

The calculated lattice energies based on the force-field energy-minimized structures are presented in the supporting information (Table S3). The RMSDs between the energies given by PBE-D3 and the two force fields, namely the COMPASS force field and the TMFF, are calculated and can be used to characterize the accuracy of force fields from the potential energy point of view. In this study, the TMFF for free base cocaine outperforms the COMPASS force field in reproducing the lattice energies of the CSP candidates, giving a lower RMSD than that of the COMPASS force field by a factor of 1.9. Both the TMFF and the COMPASS force field rank the experimental structure as number 2 by lattice energy, but the energy gap between rank 1 and the experimental structure is 0.0183 kJ mol^−1^ for the TMFF and 2.8774 kJ mol^−1^ for the COMPASS force field.

### 3.3. Molecular dynamics simulations   

**Figure 4:**
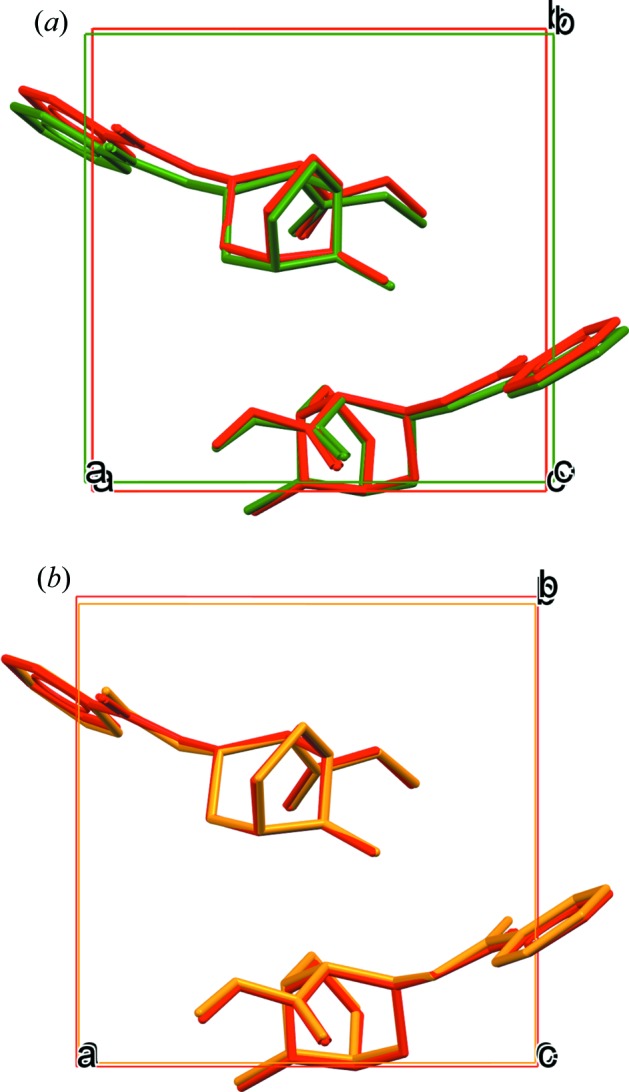
Overlays of the three-dimensional configurations of the experimental crystal structure (in red) with (*a*) the averaged structure from the MD simulation of structure 1 with the COMPASS force field (in green), and (*b*) the averaged structure from the MD simulation of structure 1 with the TMFF (in orange). Hydrogen atoms have been omitted for clarity.

Using the TMFF for free base cocaine, equilibria for the energies and cell parameters of the candidates can be reached rapidly by following the MD simulation protocol described in the *Methods* section. The TMFF was parameterized against DFT-D reference data, including some of the CSP candidates, and the CSP candidates can therefore be reproduced with the force field parameters that were fitted to them. The COMPASS force field is able to reproduce most of the structure conformations, as shown in Fig. 4 ▸. However, when the COMPASS force field was used, structures 19 and 22 showed phase transitions during the equilibration. The cell parameters of structures 11, 19 and 22 still suffered large fluctuations during their production runs. Therefore, additional 150 ps production runs were carried out and the averaged cell parameters were calculated from the additional 150 ps. The cell parameters of structure 11 still suffered large fluctuations during the additional run when the COMPASS force field was applied; therefore, we did not carry out SS-NMR calculations using motional averaging with the COMPASS force field for structure 11.

**Figure 5:**
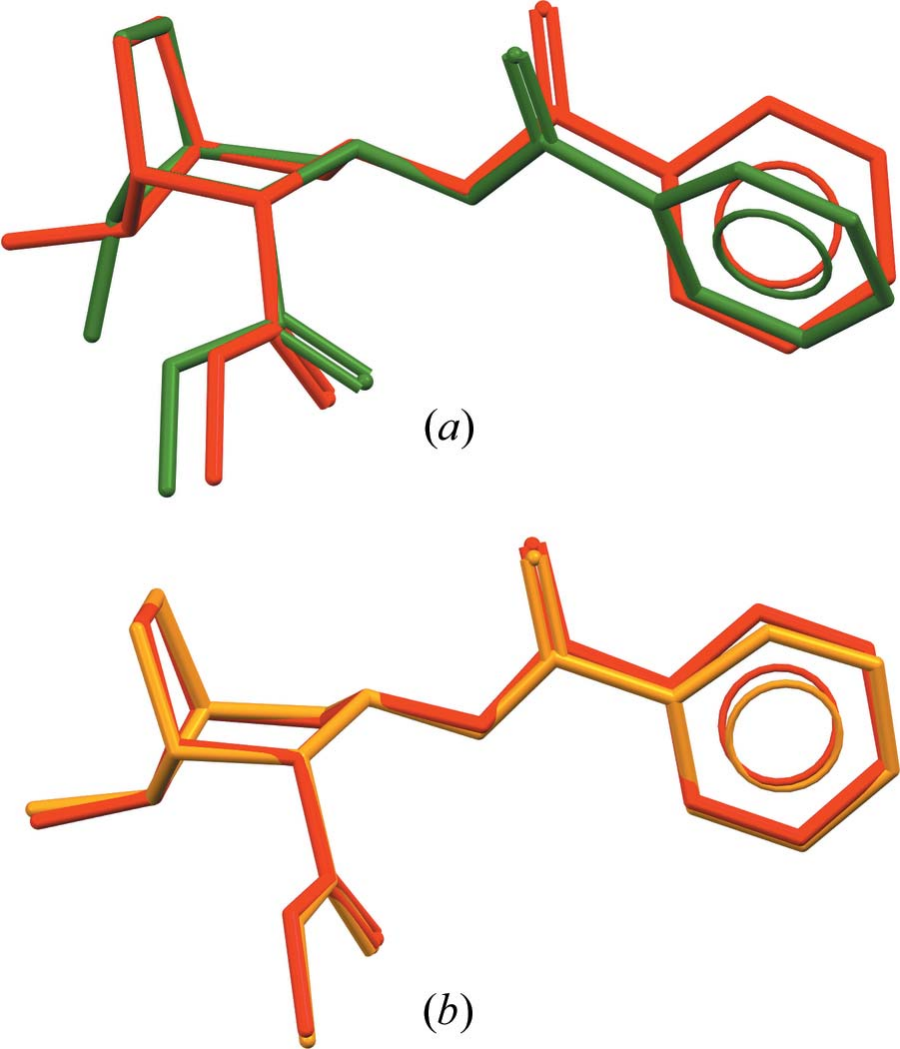
Overlays of predicted structure 19 (in red) and (*a*) the average structure calculated from the small-cell MD production run with the COMPASS force field (in green), and (*b*) the average structure calculated from the small-cell MD production run with the TMFF (in orange). Hydrogen atoms have been omitted for clarity.

Structure 19 is used here as an example of the phase transition. The variations in cell parameters and total potential energy during the MD simulations are provided in Figs. S1 and S2 in the supporting information. The average structures shown in Fig. 5 ▸ were calculated based on small-cell MD trajectories with the COMPASS force field or the TMFF. The non-H RMSCD between the averaged structure from the COMPASS force field and structure 19 from the CSP is 0.687 Å, as seen in Fig. 5 ▸(*a*). The phase transition is due to a conformational change that may be related to an inaccuracy in the potential energy given by the COMPASS force field, to a temperature effect or to both. Fig. 5 ▸(*b*) shows that the averaged structure calculated based on an MD simulation with the TMFF is very similar to the structure obtained from CSP with a rather small non-H RMSCD of 0.142 Å. The conformational change in the MD simulations with the TMFF is insignificant for all the structures that were subjected to MD simulations.

### 3.4. SS-NMR calculations: can we identify the correct structures from the calculated chemical shifts?   

#### 3.4.1. Reference shieldings   

The calculated reference shieldings of different approaches are listed in Table 1 ▸. The same protocol of magnetic shielding calculations was applied for the three different computational approaches. A noticeable difference between the ^13^C reference shieldings is given by the COMPASS force field and the TMFF of 1.5 p.p.m. This large difference indicates that, for the same system of interest, if the slope of the shift-shielding correlation is constrained to unity and the motional averaging is introduced by classical MD, the reference shielding is not only dependent upon the exchange-correlation functional used for SS-NMR calculations (Johnston *et al.*, 2009 ▸) but is also dependent upon the force field parameters used for MD simulations.

#### 3.4.2. ^13^C chemical shifts   

**Figure 6:**
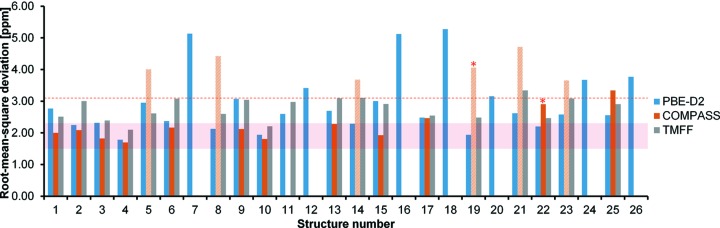
The RMSDs between the experimental and calculated ^13^C chemical shifts of the CSP candidates with experimental NMR chemical shift assignment calculated using three different computational approaches. The horizontal shaded zone indicates the calculated RMSD expectation for ^13^C chemical shifts, 1.9 ± 0.4 p.p.m. The red dashed line is located three standard deviations from the RMSD expectation (3.1 p.p.m.), which was used for selecting structures subjected to MD simulations. The bars with asterisks on the top indicate that phase transitions were identified. Bars with a paler colour indicate that the C17 methyl groups of the corresponding structures underwent an equatorial-to-axial transformation on the six-membered ring of the tropane group during the MD simulations with the COMPASS force field.

Fig. 6 ▸ shows the RMSDs between the calculated and experimental ^13^C chemical shifts for the CSP candidates. The benchmark for the calculated ^13^C RMSD, 1.9 ± 0.4 p.p.m. (indicated in Fig. 6 ▸ using a shaded zone) is obtained from Widdifield *et al.* (2016 ▸) and used as a criterion to select structures from the calculations. With this criterion, the correct experimental structure cannot be discerned: the RMSDs given by the PBE-D2, COMPASS and TMFF approaches produce seven, nine and two structures, respectively, with a confidence of one standard deviation. Structure 1 is only shortlisted through the COMPASS force field approach; the RMSDs of structure 1 given by the PBE-D2 and TMFF approaches are out of the expected RMSD range. In other words, the TMFF did not outperform the COMPASS force field approach in the aspect of averaging the ^13^C chemical shifts.

The ^13^C chemical shift calculations based on static PBE-D2 energy-minimized structures may not be sensitive enough to identify the correct form; however, the sensitivity is satisfactory to exclude structures that have a different molecular geometry than the experimental form. Examples are the extraordinarily large (≥5.0 p.p.m.) RMSDs of structures 7, 16 and 18. Indeed, these large deviations stem from the equatorial/axial conformation of the C17 methyl group (see Fig. 1 ▸). The C17 methyl group of these three candidates stands on the axial position at the six-membered ring of the tropane nitrogen, whereas the C17 methyl group predominates in the equatorial position in all other candidates. The deviations between the calculated chemical shifts of C17 in these three structures and the experimental value are larger than 9.0 p.p.m., which agrees with the difference in chemical shifts between the axial and equatorial *N*-methyl carbon atoms of tropane in experimental ^13^C solution NMR spectra (Schneider & Sturm, 1976 ▸).

When the COMPASS force field was applied for the MD simulations, conformational changes and phase transitions were discovered for several CSP candidates, as discussed in Section 3.3. Additionally, the C17 methyl groups in structures 5, 8, 14, 19, 21 and 23 changed from the equatorial to the axial position at the six-membered ring of the tropane nitrogen. The RMSDs given by the COMPASS approach were hence increased and larger than the PBE-D2 counterparts, as shown in Fig. 6 ▸. Such conformational changes were not found in the MD simulations with the TMFF.

#### 3.4.3. ^1^H chemical shifts   

**Figure 7:**
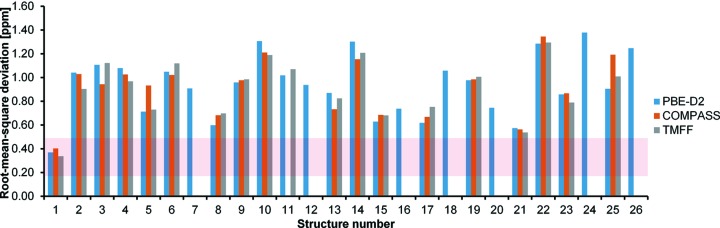
The RMSDs between the experimental and calculated ^1^H chemical shifts of the CSP candidates with experimental NMR chemical shift assignment calculated using three different computational approaches. The horizontal shaded zone indicates the calculated RMSD expectation for ^1^H chemical shifts, 0.33 ± 0.16 p.p.m.

The RMSDs between the experimental and calculated ^1^H chemical shifts for CSP candidates are shown in Fig. 7 ▸. The expectation value is 0.33 ± 0.16 p.p.m. (Widdifield *et al.*, 2016 ▸). Structure 1 is the only candidate which gives the RMSDs of all three approaches within a confidence of one standard deviation. The best match for the ^1^H chemical shifts is given by the TMFF, with an RMSD of 0.34 p.p.m..

The acquisition time for ^1^H chemical shifts in a cycle of an SS-NMR experiment is *ca* 4 ms (Taylor, 2004 ▸). This is 10^6^ times longer than the MD simulations, in which the rotation of the methyl groups has already been captured. In order to study the significance of the rotational averaging treatment for static DFT-D structures and of motional averaging with MD simulations, the differences in ^1^H chemical shift RMSDs before and after applying a single chemical shift for methyl hydrogen atoms are compared and shown in the supporting information (Table S2). As expected, the methyl groups rotate during the MD simulations, automatically averaging the ^1^H chemical shifts without any need for intervention from the user, and the RMSDs for MD simulations with either the COMPASS force field or the TMFF do not show significant variations when explicitly averaging the chemical shifts of the methyl group; the largest variation is only 0.03 p.p.m. In contrast, the influence of methyl-group averaging on RMSDs for the static DFT-D structures varies from one CSP candidate to another, ranging from 0.00 up to 0.21 p.p.m. for assigned ^1^H chemical shifts. The comparability in the RMSDs given by PBE-D2 and TMFF presented in Fig. 7 ▸ reveals that the averaging of the NMR chemical shifts over three positions for each methyl group is a practical treatment for the rotational effect, albeit in principle; the dynamics have to be introduced by *e.g.* molecular dynamics for a proper representation.

Additionally, the RMSDs do not always decrease when employing MD simulations, as seen from Fig. 7 ▸. This confirms that the improvement in RMSDs for the correct candidate is not simply because of an averaging over different atomic positions, but because of a better representation of the dynamic aspects of the atoms; with the wrong structure, including the dynamics does not make the agreement better.

#### 3.4.4. Discussion: the impact of motional averaging on ^13^C and ^1^H SS-NMR calculations   

For the ^13^C and ^1^H SS-NMR chemical shifts of proteins, it is stated that the ^13^C chemical shifts are mostly determined by the local structure, such as dihedral angles of the residues (London *et al.*, 2008 ▸; Mulder, 2009 ▸), whereas ^1^H chemical shifts are dependent on non-local intermolecular interactions, such as ring currents and hydrogen bonds (Sahakyan *et al.*, 2011 ▸; Han *et al.*, 2011 ▸). On the other hand, for packing polymorphism in organic mol­ecular crystals, the molecules are rather small and rigid and the differences in ^13^C chemical shifts are usually not obviously distinctive in different phases if the conformations of the molecules do not undergo significant changes [see, for example, the α- and the β-forms of dl-norleucine (Smets *et al.*, 2015 ▸)]. This agrees with the results obtained in this study. The correct structure can be readily discerned using ^1^H chemical shifts. The ^13^C chemical shifts provide structural details for the dihedral-angle changes related to one methyl group. This demonstrates that either a ^1^H or a ^13^C SS-NMR spectrum has its scope of applications, and therefore a combination of both spectra provides more comprehensive insight for organic molecular crystals.

The expected errors in SS-NMR calculations vary with several factors, such as the method used to predict the chemical shifts, the exchange-correlation functional used and the basis set used (Beran *et al.*, 2016 ▸). Meanwhile, this study reveals that the motional averaging introduced by different force fields has an impact on the reference shieldings. For this reason, we would expect that the force field used will also affect the errors in SS-NMR calculations. This study adopted the errors from static DFT-D structures because a statistical benchmark for motional averaging with force fields has not been established; the methodology was only applied for a single case, namely free base cocaine. One future perspective is to determine the expected errors in SS-NMR calculations based on motional averaging with a test set of molecular crystals.

A major advantage of the TMFF over the COMPASS force field is that the DFT-D energy minima remains stable during the MD simulations. In other words, the TMFF provides a better description of the dynamics of the CSP candidates as neither a phase transition nor a significant conformational change was discovered. With such a high-quality force field, the improvement in the accuracy of NMR calculations does not seem to be significant. It is, in fact, unsurprising because isotropic shieldings are not as sensitive to conformational change as anisotropic shieldings. For example, Liu *et al.* (1995 ▸) calculated the variation in principal values and isotropic chemical shieldings of atom C1 against the change in the dihedral angle H[O1]—O1—C1—C2 in *meso*-erythritol using the gauge-invariant atomic orbital (GIAO)-based method. They discovered that the variations in the δ_11_ and δ_22_ principal values were greater than 10 p.p.m. during rotation, whereas the isotropic chemical shift δ_iso_ only varied by *ca* 2 p.p.m. Therefore, it is appealing to study the agreement between calculated and experimental anisotropic chemical shifts, if such experimental data are available.

In the meantime, a recent investigation of the ^1^H, ^13^C, ^15^N and ^17^O chemical shifts for four benchmark sets of molecular crystals shows that the use of fragment-based electronic structure methods coupled with hybrid functionals out­performed the GIPAW method, which is commonly used with GGA functionals such as PBE (Hartman *et al.*, 2016 ▸). According to this investigation, the expected RMSD of ^13^C chemical shifts given by the charge-embedded two-body fragment method and the PBE0 functional (Perdew *et al.*, 1996 ▸; Adamo & Barone, 1999 ▸) is 1.5 p.p.m. for static molecular crystals, which is better than both the DFT-GIPAW obtained from the same study (2.2 p.p.m.) and the expectation used in this study (1.9 p.p.m.; Widdifield *et al.*, 2016 ▸). This investigation and our study indicate that, although motional averaging is able to lessen the errors in NMR calculations, it pales into insignificance in comparison with the intrinsic error in the DFT-GIPAW method with the plane-wave implementation, in which the use of a hybrid functional requires at least an order of magnitude more computing power.

## 4. Conclusions   

The performance of a TMFF for free base cocaine for the motional averaging aspects of *ab initio* ^13^C and ^1^H isotropic NMR chemical shift calculations is assessed and compared with existing benchmarks, including static PBE-D2 energy minimizations (Li *et al.*, 2014 ▸), motional averaging with the COMPASS force field (Li *et al.*, 2016 ▸) and the literature (Baias, Widdifield *et al.*, 2013 ▸). In general, the TMFF gives an accurate representation for the motional averaging of CSP candidates, but this does not give a significant improvement in the accuracy of SS-NMR calculations.

The TMFF of free base cocaine is parameterized against DFT-D reference data based on the crystal packings of cocaine. The reproduction of the lattice energies of CSP candidates using the TMFF is superior to the COMPASS force field. During the MD simulations, the equilibria can be reached rapidly with the TMFF; no phase transition was observed. On the other hand, unexpected conformational changes and phase transitions were captured for a few candidates in this study when the COMPASS force field was applied. This may be connected with the preferred energy minima of the COMPASS force field, or to the effect of temperature.

Earlier case studies reveal that the deviations between the calculated and experimental ^13^C chemical shifts from static structures are not able to discern the correct structure from a list of generated crystal structure candidates (usually from crystal structure prediction) (Baias, Widdifield *et al.*, 2013 ▸). The motional averaging introduced by classical MD with either the COMPASS force field or the TMFF is not able to resolve the crystal structure. The ^13^C chemical shifts are closely related to the local structure, *i.e.* the molecular geometry, whereas cocaine polymorphs have similar conformations, which is a factor that increases the difficulty of structure selection based on the ^13^C chemical shifts. For crystalline forms that exhibit conformational polymorphism because of molecular flexibility, the evaluation of ^13^C chemical shifts still plays an important role. In this particular case for free base cocaine, ^13^C chemical shifts show a good performance in distinguishing the axial and equatorial conformations of the *N*-methyl carbon in the tropane group.

Combining experimental ^1^H SS-NMR spectroscopy with calculations, in most of the cases, has been shown to be a robust method of identifying the correct crystal structure candidate (Salager *et al.*, 2010 ▸; Baias, Widdifield *et al.*, 2013 ▸). Indeed, ^1^H chemical shifts are firmly related to intermolecular interactions such as hydrogen bonds. Both packing polymorphism and conformational polymorphism are mostly dominated by a range of intermolecular forces, and this explains the capability of ^1^H SS-NMR spectra in NMR crystallography for molecular crystals.

This study has examined and discussed the limit of motional averaging in the calculation of ^1^H and ^13^C isotropic chemical shifts. First, introducing motional effects has little impact on the isotropic chemical shifts. Second, the chemical shifts were all calculated using the GIPAW method, no matter which force field was applied to sample the thermal motion. The intrinsic errors in the GIPAW method are much more significant than the improvement brought by motional averaging. In order to overcome such limitations, we suggest drawing more attention to the study of anisotropic chemical shifts using both calculation and experiment, and improvement of the methods for NMR chemical shift prediction.

## Supplementary Material

Additional tables and figures. DOI: 10.1107/S2052252517001415/ct5003sup1.pdf

CIF data for simulations. DOI: 10.1107/S2052252517001415/ct5003sup2.txt

## Figures and Tables

**Table 1:** The reference shieldings of different computational approaches The reference shielding calculated from five ^13^C SS-NMR calibration phases is listed here for comparison.

	Reference shielding (p.p.m.)	
Computational approach	^1^H	^13^C
PBE-D2	31.17	168.8
COMPASS	30.99	169.4
TMFF	30.98	167.9
Five ^13^C calibration phases (Li *et al.*, 2016 ▸)	N/A	168.9
