# Supplementary material for: The application of tailor-made force fields and molecular dynamics for NMR crystallography: a case study of free base cocaine
Source: IUCrJ. 2017 Feb 15;4(Pt 2):175–84. doi: 10.1107/S2052252517001415 (PMC5330528; doi:10.1107/S2052252517001415)
Supplement: Supplementary file 1 [file m-04-00175-sup1.pdf]

IUCrJ

Volume 4 (2017)

Supporting information for article:

The application of tailor-made force fields and molecular dynamics for NMR crystallography: a case study of free base cocaine

Xiaozhou Li, Marcus A. Neumann and Jacco van de Streek

Table S1 The structure information of the predicted structures of free base cocaine.

Rank	Energy [kJ/mol]	Density [g/cm^3]	Volume [Å^3]	Space group	*a* [Å]	*b* [Å]	*c* [Å]	α [degree]	β [degree]	γ [degree]
1	-25831.6969	1.2774	788.7195	$P2_1$	8.3957	9.6583	10.1543	90	73.3123	90
2	-25827.2653	1.2944	778.3277	$P2_1$	6.6664	14.3226	8.1670	90	86.5023	90
3	-25826.0714	1.2746	1580.9005	$P2_12_12_1$	9.7195	11.1644	14.5689	90	90	90
4	-25824.9545	1.2890	1563.2394	$P2_12_12$	12.8938	14.6823	8.2575	90	90	90
5	-25824.7340	1.2618	1596.8403	$P2_12_12_1$	9.2611	10.7589	16.0262	90	90	90
6	-25824.6800	1.2737	1581.9747	$C2$	14.1211	9.7160	12.9936	90	117.4531	90
7	-25823.4961	1.2958	1554.9391	$P2_12_12_1$	9.2184	10.1863	16.5592	90	90	90
8	-25822.6415	1.2689	1588.0045	$P2_12_12_1$	8.3945	8.9344	21.1733	90	90	90
9	-25822.2282	1.2647	398.3078	$P1$	5.7560	7.2550	10.1966	99.7982	73.9437	101.2150
10	-25822.1247	1.2945	1556.5633	$P2_12_12_1$	9.6637	11.3606	14.1782	90	90	90
11	-25821.3052	1.2511	1610.5589	$P4_1$	10.3079	10.3079	15.1578	90	90	90
12	-25821.1311	1.3131	1534.4899	$P2_12_12_1$	6.6666	14.1215	16.2995	90	90	90
13	-25821.0690	1.2499	806.0592	$P2_1$	5.7789	14.3995	9.9849	90	75.9637	90
14	-25821.0031	1.2930	1558.3879	$P2_12_12_1$	8.3389	10.7827	17.3315	90	90	90
15	-25820.7577	1.2979	1552.4772	$P2_12_12$	9.7151	19.3682	8.2506	90	90	90
16	-25820.6289	1.3032	1546.1436	$P2_12_12_1$	8.4011	9.0823	20.2635	90	90	90
17	-25820.5621	1.2896	1562.4933	$P2_12_12_1$	9.8101	12.8048	12.4386	90	90	90
18	-25820.3971	1.2839	1569.4381	$P2_12_12_1$	9.3468	10.8756	15.4394	90	90	90
19	-25819.5549	1.2299	819.1681	$P2_1$	8.1573	7.3473	13.8744	90	99.9022	90
20	-25819.4253	1.2808	786.5962	$P2_1$	7.0409	9.8919	11.8351	90	107.3928	90
21	-25818.8189	1.3000	775.0017	$P2_1$	8.2680	10.8413	9.4488	90	113.7877	90
22	-25818.4618	1.2740	790.8067	$P2_1$	9.7748	7.1420	12.2300	90	67.8537	90
23	-25818.3217	1.2910	1560.7262	$P2_12_12_1$	6.9114	10.2164	22.1036	90	90	90
24	-25817.7597	1.2769	1578.0043	$P2_12_12_1$	8.2919	8.5583	22.2365	90	90	90
25	-25816.9300	1.2635	1594.6769	$P2_12_12_1$	7.0886	8.6926	25.8801	90	90	90
26	-25816.6015	1.3297	757.6665	$P2_1$	7.7978	13.0465	7.9215	90	70.0799	90

Figure S1 The changes of the cell parameters of structure 19 during the MD simulation using the COMPASS force field. (a): The cell lengths. (b) The cell angles.

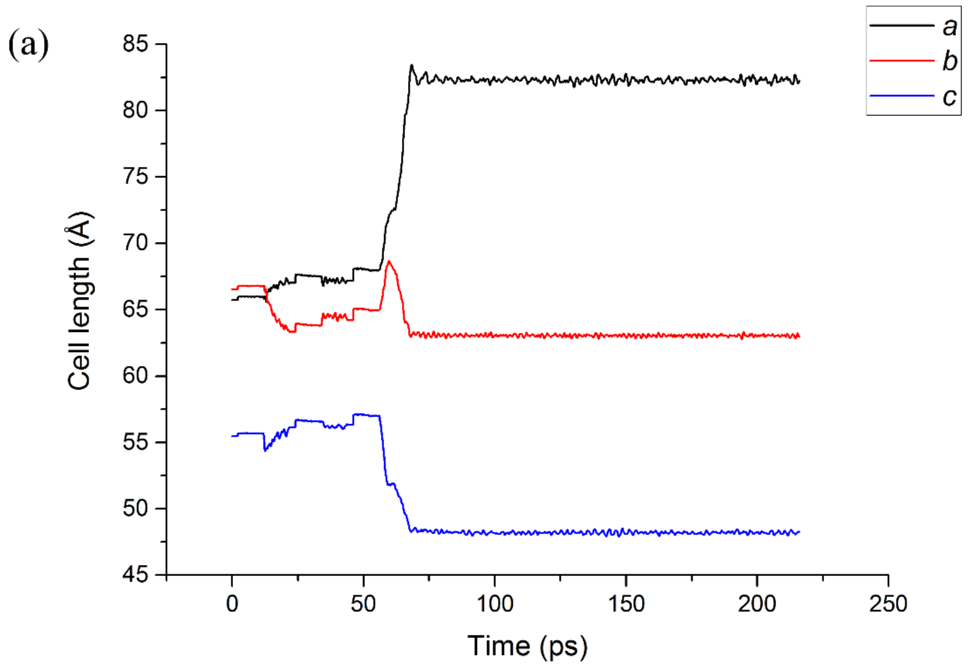

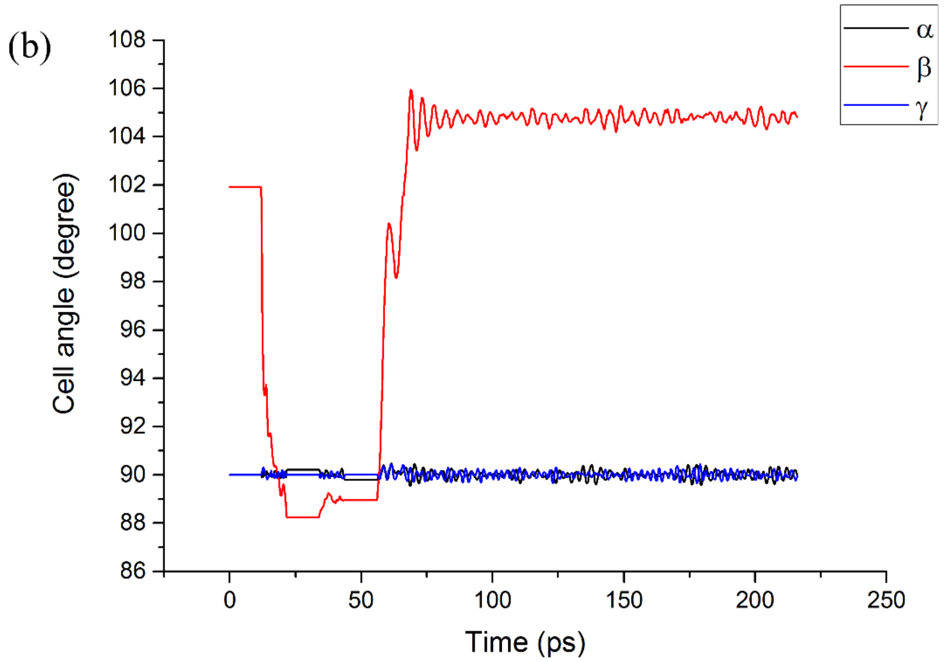

Figure S2 The change of the total potential energy of structure 19 during the MD simulation using the COMPASS force field.

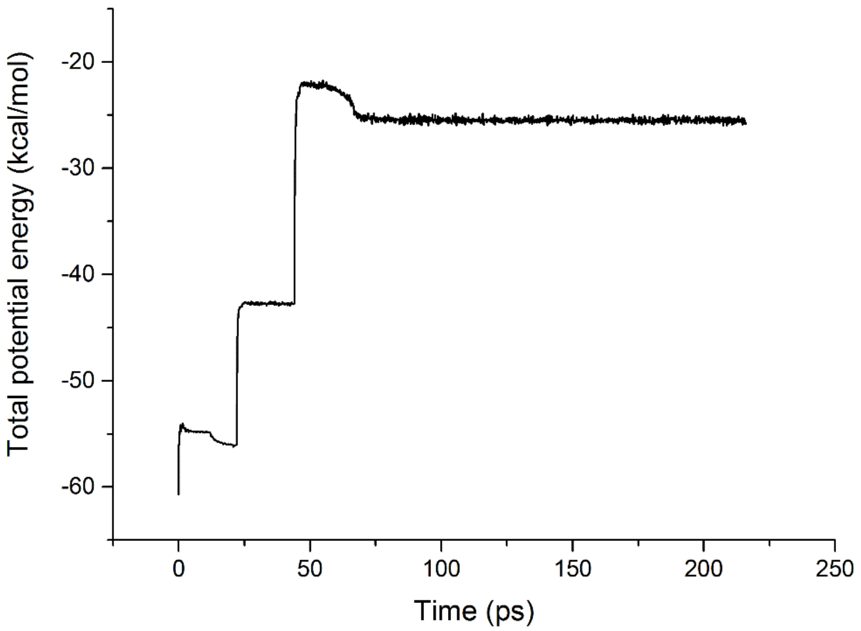

Table S2 The root-mean-square deviations (RMSDs) between the calculated and the experimental ^{1}H chemical shifts with assignment. “Single value” refers to using one averaged chemical shift for the hydrogen atoms on each methyl group. “Multiple values” refers to using individually calculated chemical shifts for the hydrogen atoms on each methyl group.

CSP Candidate	PBE-D2			COMPASS			TMFF		
	Single value [ppm]	Multiple values [ppm]	Absolute deviation [ppm]	Single value [ppm]	Multiple values [ppm]	Absolute deviation [ppm]	Single value [ppm]	Multiple values [ppm]	Absolute deviation [ppm]
1	0.37	0.52	0.15	0.40	0.42	0.02	0.34	0.34	0.00
2	1.04	1.08	0.04	1.03	1.03	0.00	0.90	0.91	0.01
3	1.11	1.14	0.03	0.94	0.94	0.00	1.12	1.12	0.00
4	1.08	1.15	0.07	1.03	1.03	0.00	0.97	0.97	0.00
5	0.71	0.81	0.10	0.93	0.93	0.00	0.73	0.73	0.00
6	1.05	1.06	0.01	1.02	1.02	0.00	1.12	1.12	0.00
7	0.91	0.92	0.01						
8	0.60	0.66	0.06	0.68	0.69	0.01	0.70	0.70	0.00
9	0.96	0.99	0.03	0.98	0.98	0.00	0.98	0.99	0.01
10	1.31	1.31	0.01	1.21	1.21	0.00	1.19	1.19	0.00
11	1.02	1.04	0.02				1.07	1.07	0.00
12	0.94	0.94	0.00						
13	0.87	0.91	0.04	0.73	0.74	0.01	0.82	0.83	0.01
14	1.30	1.33	0.03	1.15	1.15	0.00	1.21	1.21	0.00
15	0.63	0.65	0.02	0.69	0.69	0.00	0.68	0.68	0.00
16	0.74	0.85	0.11						
17	0.62	0.83	0.21	0.67	0.67	0.00	0.75	0.76	0.01
18	1.06	1.09	0.03						
19	0.98	1.00	0.02	0.98	0.99	0.01	1.01	1.01	0.00
20	0.74	0.78	0.04						
21	0.57	0.72	0.15	0.56	0.57	0.01	0.54	0.54	0.00
22	1.29	1.32	0.03	1.34	1.34	0.00	1.29	1.30	0.01
23	0.86	0.88	0.02	0.87	0.87	0.00	0.79	0.79	0.00
24	1.38	1.39	0.01						
25	0.91	0.99	0.08	1.19	1.20	0.01	1.01	1.01	0.00
26	1.25	1.27	0.02						

Table S3 The relative lattice energies given by energy minimised structures using PBE-D3, the COMPASS force field and the TMFF. In the "Relative lattice energy" column, the average lattice energy of all 26 candidates given by each method was calibrated to zero. The RMSDs between PBE-D3 and the two force fields are provided for a quantitative comparison.

CSP Candidate	Relative lattice energy [kJ/mol]						
	PBE-D3	COMPASS	Deviation (COMPASS)	Rank (COMPASS)	TMFF	Deviation (TMFF)	Rank (TMFF)
1	-10.0577	-8.5782	1.4795	2	-4.9826	5.0751	2
2	-5.6261	-4.0078	1.6183	10	-3.2749	2.3512	4
3	-4.4321	-6.1491	-1.7170	5	-0.8151	3.6170	12
4	-3.3153	-4.0911	-0.7758	9	-3.4565	-0.1412	3
5	-3.0948	1.5974	4.6922	19	3.7073	6.8021	22
6	-3.0407	-8.2715	-5.2308	3	-5.0009	-1.9602	1
7	-1.8569	-6.1046	-4.2477	6	-0.3775	1.4794	15
8	-1.0022	-0.4768	0.5254	14	0.0111	1.0133	17
9	-0.5890	-0.7063	-0.1173	12	0.1477	0.7367	18
10	-0.4854	-2.6460	-2.1606	11	-0.5732	-0.0878	14
11	0.3340	11.5374	11.2034	24	-1.2005	-1.5345	9
12	0.5082	0.0874	-0.4208	15	0.1889	-0.3193	19
13	0.5703	1.3103	0.7400	18	-0.8659	-1.4362	11
14	0.6362	2.8861	2.2499	20	-0.6412	-1.2774	13
15	0.8815	-6.3781	-7.2596	4	-1.9341	-2.8156	7
16	1.0104	-11.4556	-12.4660	1	1.5031	0.4927	20
17	1.0772	-5.9480	-7.0252	8	-2.9787	-4.0559	5
18	1.2421	-6.1045	-7.3466	7	-2.8931	-4.1352	6
19	2.0844	16.1418	14.0574	26	7.0601	4.9757	26
20	2.2139	12.5006	10.2867	25	2.8628	0.6489	21
21	2.8203	1.1943	-1.6260	17	-0.9372	-3.7575	10
22	3.1775	-0.4993	-3.6768	13	-1.5194	-4.6969	8
23	3.3176	5.9433	2.6257	21	5.9440	2.6264	24
24	3.8796	9.2684	5.3888	23	6.2577	2.3781	25
25	4.7093	7.8532	3.1439	22	3.9908	-0.7185	23
26	5.0377	1.0965	-3.9412	16	-0.2227	-5.2604	16
RMSD [kJ/mol]			5.8979			3.0899	
